# Breast-conserving surgery in locally advanced breast cancer submitted to neoadjuvant chemotherapy. Safety and effectiveness based on ipsilateral breast tumor recurrence and long-term follow-up

**DOI:** 10.6061/clinics/2017(03)02

**Published:** 2017-03

**Authors:** Guilherme Freire Angotti Carrara, Cristovam Scapulatempo-Neto, Lucas Faria Abrahão-Machado, Maria Mitzi Brentani, João Soares Nunes, Maria Aparecida Azevedo Koike Folgueira, René Aloisio da Costa Vieira

**Affiliations:** I Hospital de Câncer de Barretos, Programa de Pós-Graduação em Oncologia, Barretos/SP, Brazil; II Hospital de Câncer de Barretos, Departamento de Patologia, Barretos/SP, Brazil; III Faculdade de Medicina da Universidade de São Paulo, Disciplina de Oncologia, Departamento de Radiologia, São Paulo/SP, Brazil; IV Hospital de Câncer de Barretos, Departamento de Oncologica Clínica, Barretos/SP, Brazil; V Hospital de Câncer de Barretos, Programa de Pós-Graduação em Oncologia, Departamento de Mastologia e Reconstrução Mamária, Barretos/SP, Brazil

**Keywords:** Breast Neoplasms, Neoadjuvant Therapy, Drug Therapy Combination, Breast-Conserving Surgery, Recurrence, Disease-Free Survival

## Abstract

**OBJECTIVE:**

To evaluate ipsilateral breast tumor recurrence after breast-conserving surgery for locally advanced breast cancer.

**METHODS:**

A retrospective observational cohort study was performed in patients with locally advanced breast cancer submitted to breast-conserving surgery after neoadjuvant chemotherapy based on an adriamycin-cyclophosphamide-paclitaxel regimen. We evaluated the clinical, pathologic, immunohistochemistry, and surgical factors that contribute to ipsilateral breast tumor recurrence and locoregional recurrence. A Kaplan-Meier analysis and Cox model were used to evaluate the main factors related to disease-free survival.

**RESULTS:**

Of the 449 patients who received neoadjuvant chemotherapy, 98 underwent breast-conserving surgery. The average diameter of the tumors was 5.3 cm, and 87.2% reached a size of up to 3 cm. Moreover, 86.7% were classified as clinical stage III, 74.5% had T3-T4 tumors, 80.5% had N1-N2 axilla, and 89.8% had invasive ductal carcinoma. A pathologic complete response was observed in 27.6% of the tumors, and 100.0% of samples had free margins. The 5-year actuarial overall survival rate was 81.2%, and the mean follow-up was 72.8 months. The rates of ipsilateral breast tumor recurrence and locoregional recurrence were 11.2% and 15.3%, respectively. Multifocal morphology response was the only factor related to ipsilateral breast tumor recurrence disease-free survival (*p*=0.04). A multivariate analysis showed that the pathologic response evaluation criteria in solid tumors (RECIST)-breast cutoff was the only factor related to locoregional recurrence disease-free survival (*p*=0.01).

**CONCLUSIONS:**

Breast-conserving surgery is a safe and effective therapy for selected locally advanced breast tumors.

## INTRODUCTION

Breast cancer is the most common cancer among women. Approximately 1.7 million new cases are estimated to occur worldwide, and mortality is increasing in developing countries, primarily because the disease is not diagnosed until it is in an advanced stage 1. In the past decade, advanced stage III and IV carcinomas represented 8.5% of tumors in United States and 44.7% of tumors in Brazil, making advanced carcinoma a public health problem in the latter country 2.

From clinical, biological and pathologic perspectives, locally advanced breast cancer (LABC) represents a relatively heterogeneous group of tumors. Although neoadjuvant chemotherapy (NC) does not increase the survival rates, it is used to improve tumor resection, increase the rates of breast-conservative surgery (BCS) 3,4, and identify patients with better prognoses, that is, patients who exhibit a pathologic complete response (pCR) 5.

The rate of conservative surgery after NC varies from 37% to 82% 6,7; however, only 1.7% to 28% of these cases are classified as LABC 7,8. The role of conservative surgery in the treatment of breast cancer is well established if the surgery is combined with radiotherapy 5,9. Studies of large cohorts of patients with LABC who underwent NC and conservative surgery are limited 10. The safety of BCS may be assessed based on the rates of local and locoregional recurrence (LRR). The selection of patients for BCS depends on the tumor characteristics, pre- and post-chemotherapy clinical-radiologic correlation, chemotherapy type, the marking and resection of the tumor bed, excision margins, the type of response to chemotherapy, and the molecular subtype 11-14. Local recurrence and LRR are influenced by the tumor characteristics, the size of the initial or residual tumor, the rate of initial or residual lymph node metastatic disease, the type of response to NC, the duration of follow-up, the expression of markers measured by immunohistochemistry, and the molecular subtype 11-15. However, studies of large cohorts are necessary to assess the safety of BCS in patients with LABC subjected to the same chemotherapy regimen. These studies should evaluate the clinical, pathologic, and molecular factors associated with ipsilateral breast tumor recurrence (IBTR) and LRR.

## MATERIALS AND METHODS

This study examined a retrospective observational cohort of sequential patients with a clinical diagnosis of non-metastatic LABC who had not undergone previous treatment but received NC and BCS at an oncology tertiary hospital between October 2005 and December 2012. LABC was defined as patients with clinical stage III disease, i.e., advanced tumors at diagnosis with tumors larger than 5 cm, clinical N2 metastasis, clinical skin infiltration, or clinical “peau d’orange” at diagnosis. Of the 449 patients subjected to NC, 98 received BCS and were included in the study. Patients who underwent a mastectomy were excluded from the analysis. Patients were selected for BCS based on tumor size, breast-tumor relation, clinical aspects, response to NC, radiological and post-NC evaluation and surgeon expertise. T3 tumor selection was based breast-tumor relation, associated with free surgical margins. Only localized clinical T4 tumors were selected for BCS. Inflammatory breast cancer was not selected for BCS. Two patients who initially underwent BCS had intraoperative positive margins and received a mastectomy. Thus, they were excluded from the analysis. Table 1 presents the differences between the groups of surgeries performed.

At the time of the observation, the standard NC regimen consisted of 4 cycles of AC (doxorubicin [adriamycin] 60 mg/m^2^ + cyclophosphamide 600 mg/m^2^), followed by 4 cycles or 12 cycles of T (Taxol® [paclitaxel] 175 mg/m^2^ or 80 mg/m^2^, respectively). Standard Brazilian surgical treatment consisted of quadrantectomy combined with level III axillary lymph node dissection, and all patients were subjected to whole-breast adjuvant radiotherapy (5040 cGy) combined with a boost to the surgical bed (1000 cGy). They eventually also received radiotherapy to the supraclavicular fossa. Axillary radiotherapy was not performed. Five-year adjuvant hormonal therapy was indicated for patients with estrogen/progesterone-receptor (ER/PR)-positive tumors. No additional chemotherapy was administered after surgery and was only indicated as a palliative treatment in cases of recurrence. Neoadjuvant trastuzumab was not administered, and adjuvant trastuzumab was provided to some patients.

Clinical, surgical, and histological data as well as tumor marker expression were assessed using immunohistochemistry; all data were standardized. For bilateral tumors, the tumors exhibiting the most advanced stage were considered. The tumor slides were assessed by pathologists (CSN and LFAM), and pathologic characteristics were based on open biopsy specimen at the time of diagnosis. The tumor, node, metastasis (TNM) clinical staging system (7^th^ edition, 2010) was used. Prior to chemotherapy, clinical and radiologic staging and systematic breast assessment were performed for all patients. These clinical criteria were used to identify LABC. If radiologic evaluation resulted in down-staging, the patients were considered for analysis, but clinical-breast radiologic evaluation defined the staging. The classification of the morphologic response formulated by Chen et al. 11 from MD Anderson Cancer Center (MDA) was used to classify the pathologic response to chemotherapy in the breast, which is based on the response of the solid mass, residual multifocal disease, and no residual tumor (absence of tumor and in situ carcinoma). Stable disease was added to this classification, and we considered it a morphologic classification. The response evaluation criteria in solid tumors (RECIST) radiologic classification cutoff values were adapted for 1-dimensional pathologic breast assessment [(RECIST-breast (RECIST-B)], with the cutoff points for 1-dimensional invasive breast disease established as 30% for partial response and 10% for progressive disease 16. A pCR was defined as the absence of invasive disease in the breast and axilla.

### Follow-up

The patients were selected based on clinical and radiologic aspects before and after NC. The selected patients were subjected to surgery, which was performed by a team comprising 5 surgical oncologists and 1 breast cancer specialist. The team’s preferential approach was the resection of the entire disease area prior to NC 17. The planning was based on tumor marking performed prior to NC, clinical assessment, and radiologic assessment before and after NC. The frozen section analyses were available during the surgeries. All patients were assessed by multidisciplinary staff during the postoperative period.

The patients were assessed every 6 months for 5 years, and then once per year for an additional 5 years. The total follow-up period was counted from the first visit to the last available day. Breast clinical and radiological annual evaluations were performed. In the presence of clinical signs, symptoms, or proven recurrence, the patient was submitted for new radiological staging. The time between the first evaluation to the surgery was evaluated. Disease-free survival (DFS) was defined as the time elapsed from the performance of quadrantectomy to either the recurrence of disease or the last follow-up visit. All patients who missed 2 scheduled visits were considered lost to follow-up.

### Endpoints

The response to chemotherapy, IBTR, and LRR were assessed. IBTR was defined as a local relapse of breast cancer, even in the case of secondary breast local invasion. LRR was defined as local relapse associated with ipsilateral regional lymph node disease. Metachronous contralateral breast tumors were not considered to be recurrences. In the presence of recurrence, the patients were submitted to thoracic and abdominal tomography associated with bone scintigraphy.

### Molecular subtypes

Systematic assessment of samples in 10% neutral buffered formalin blocks was performed using a tissue microarray (TMA). The approximate molecular subtypes were assessed by immunohistochemistry. The tumor markers anti-ER SP1, anti-PR 1E2, anti-HER2/neu (clone 4B5), and anti-Ki-67 (clone 30-9) were used (Roche Diagnostics). Tumors were rated ER/PR-positive when nuclear labeling was evident in more than 1% of tumor cells. A semi-quantitative score was used for HER2 staining. A score of 0, 1+, or 2+ was considered negative staining, and a score of 3+ was considered positive staining. Fluorescence in situ hybridization (FISH) was performed for samples with a 2+ score. The approximate molecular subtypes were clustered as a function of the immunohistochemical results as follows: luminal A (ER/PR-positive, HER2-negative, and Ki-67<14), luminal B/HER-negative (ER/PR-positive, HER2-negative, and Ki-67 ≥14), luminal B/HER-positive (ER/PR-positive and HER2-positive), HER2 (ER/PR-negative and HER2-positive), and triple negative (ER/PR-negative and HER2-negative). In the absence of TMA information, the primary immunohistochemical lamina was reviewed. Discrepancies were discussed until reaching consensus. To evaluate the analysis, luminal A and luminal B/HER-negative samples were grouped into the luminal/HER-negative group.

### Statistical analysis

The data were collected, standardized, tabulated, and analyzed using the SPSS 20.0 software for Mac® (Armonk, New York, NY). Univariate analyses of the categorical variables related to local and locoregional DFS were performed using the Kaplan-Meier method. The difference between the curves was assessed with the log-rank method. A Cox model was used to identify variables independently associated with local and locoregional DFS. We evaluated continuous variables without dichotomization. For Cox modeling, we evaluated categorical interest variables and variables exhibiting *p*<0.10 in the univariate analysis. An exploratory model was used for Cox multivariate analyses. The significance level was set to *p*<0.05.

### Ethics

The study was approved by the Research Ethics Committee, no. 135/2008.

### STROBE statement

This study adhered to the STROBE guidelines for cohort studies.

## RESULTS

Ninety-eight patients with LABC who had undergone NC and BCS were evaluated. Tumor size, clinical TNM stage, pT-TNM, and pN-TNM were lower, whereas the incidence of triple-negative tumors was higher in the BCS group than the mastectomy group; age did not significantly differ between these groups (Table 1). The average age of patients who had undergone BCS was 48.5 years old, and the average duration of complaint was 8 months. Bilateral tumors were identified in 2% of samples. The average diameter of the tumors was 5.3 cm (2 to 8.5 cm). Clinical-radiologic staging was performed for all patients, but 4 patients (4.2%) were classified as stage IIa (T2N0) after initial radiologic examination and were further analyzed. Bone scintigraphy was performed for all patients. In 58.2% of patients, staging was based on chest radiographs and abdominal ultrasound, whereas it was based on thoracic and abdominal computed tomography in the remaining patients. All patients underwent mammography with breast ultrasound (64.3%) or ultrasound and magnetic resonance imaging (MRI) (23.5%). Lesions were preoperatively marked on the skin in 23.5% patients. The main characteristics related to tumor staging and treatment are described in Table 2.

Chemotherapy treatment included the 4AC+4T (81.6%) and 4AC+12T (11.2%) regimens, and the standard regimen was modified in 7.1% of patients because of toxicity (4.1%) or disease progression (3.1%).

### Surgery

Surgery was performed 43 days after the end of chemotherapy, on average. The average duration from the first visit to surgery was 8.3 months. All patients underwent a quadrantectomy, and oncoplastic surgery was performed in 26.5% of patients, which was distributed as follows: central quadrantectomy (8.1%), rotation flap (7.1%), periareolar (5.1%), inferior pedicle (4.1%), and superior pedicle (2.0%). Level III axillary lymph node dissection was performed in 97.0% of patients, the sentinel lymph node was investigated in 2.0% of patients, and no axillary approach was employed in 1.0% of patients.

The margins were tumor-free in all patients, and 81.6% of patients harbored tumors measuring 12.3 mm (1 to 40 mm) on average. Moreover, 13.3% of patients had pCR, and the margins were not evaluated. In 5.1% of patients 5, the margins were considered free, and the distance measurement was not evaluated. The average weight of the surgical specimens was 233 g (41.5 to 980 g). The average number of dissected lymph nodes reported during pathologic evaluation was 18.5 (4 to 42). Table 3 presents the response to NC.

### Adjuvant treatment

With regard to adjuvant therapy, 98% of patients received radiotherapy to the chest wall (5040 cGy) and a boost to the breast (1000 cGy) near the incision. Radiotherapy to the supraclavicular fossa was performed in 89.4% of patients. Two patients did not receive radiotherapy, 1 because of rapid disease progression and the other refused because of claustrophobia. Hormonal therapy was administered to 57.1% of patients and consisted of tamoxifen alone, anastrozole alone, or a combined regimen in 35.7%, 3%, and 18.4% of patients, respectively. Adjuvant trastuzumab was administered to only 2.0% of patients.

### Follow-up

The average duration of follow-up was 64.1 months (13.4 to 105.7 months), with the follow-up period decreasing to 55.8 months (3.6 to 95.7 months) after surgery. After the exclusion of deaths due to disease, the average total duration of follow-up was 72.8 months (34.4 to 105.7 months) and 64.1 months (26.3 to 95.9 months) after surgery. Moreover, 6.1% of patients 6 were considered lost of follow-up, and 5/6 patients exhibited DFS with a mean time of 39.4 months (26.3-48.6 months).

**Figure 1:**
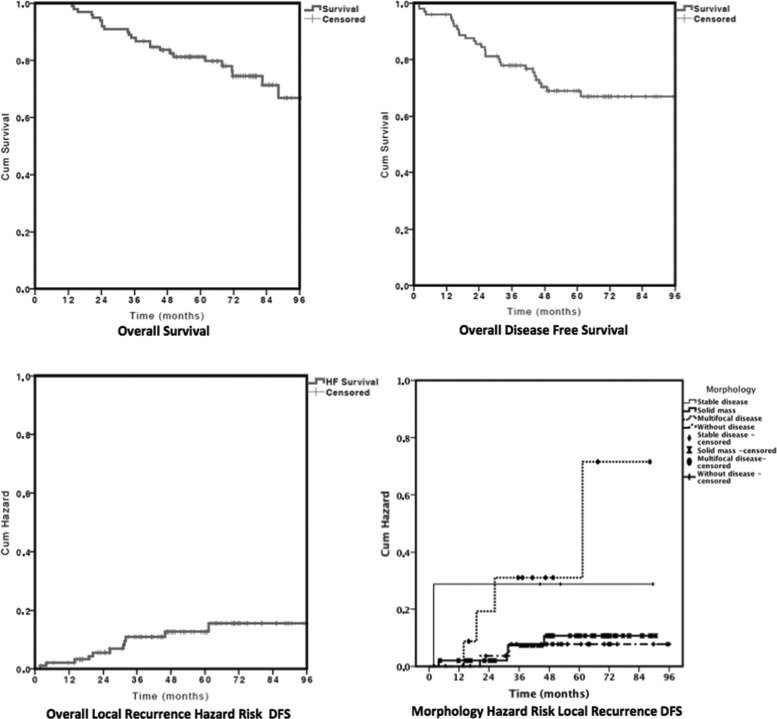
OS of patients with NC and BCS (upper images) and hazard risk of local recurrence DFS (lower images).

By the end of the follow-up period, 19.5% of the patients had died from breast cancer, 5.1% had died from other causes, 7.1% were living with cancer, and 68.4% were alive and free of disease. The main sites of metastases were in the bones (16.3%), lungs (13.3%), liver (8.2%), and brain (4.1%). The overall actuarial survival (OS) rates at 36, 60, and 96 months were 87.7%, 81.2%, and 71.4%, respectively (Figure 1).

### Recurrence

The average months after surgery for recurrence vary from 1.8 to 81.3 months and was 26.4, 26.8 and 27.1 months in general, IBTR and LRR, respectively. At 36 months 72.4% of the general recurrence occurred, 83.3% of the IBTR and 76.5% of the LRR. The overall DFS rates at 36, 60 and 96 months were 77.9%, 68.9% and 67.0%, respectively (Figure 1).

The IBTR rate was 11.2%. IBTR was classified as local recurrence associated with systemic disease (3.1%), local plus LRR (3.1%), breast recurrence alone (3.1%), and secondary breast invasion from sternal recurrence (2.0%). When assessing disease progression, some patients exhibited rapidly progressive disease with early local recurrence plus LRR (2.0%), sternal recurrence extending to the breast (2.0%), breast recurrence alone (3.1%), and multiple local recurrence plus LRR (4.1%). Excluding the instance of local sternal recurrence that infiltrated the breast, the primary recurrence rate was 9.3%.

A univariate analysis of local DFS relative to the categorical variables (Table 1) showed that the absence of necrosis (*p*=0.04) and the morphologic response to chemotherapy characterized by multifocal disease and stable disease were associated with poorer survival (*p*=0.04). Neither age [*p*=0.33, risk ratio 1.01, confidence interval (CI) 0.99-1.03] nor initial tumor size (*p*=0.35, risk ratio 1.06, CI 0.93-1.20) influenced IBTR DFS. The Cox univariate analysis, which was used to explore factors possibly related to IBTR DFS (Table 2), showed that multifocal morphology was the only factor associated with IBTR because it increased the IBTR 5.97-fold (*p*=0.04). Figure 1 shows the overall and morphology factor risk curves related to the hazard risk of local DFS.

The LRR rate was 15.3% and was distributed as follows: ipsilateral supraclavicular fossa (4.0%), ipsilateral axilla (3.1%), local and systemic recurrence (3.1%), breast recurrence alone (3.1%), and breast associated with sternal recurrence (2.0%).

**Figure 2:**
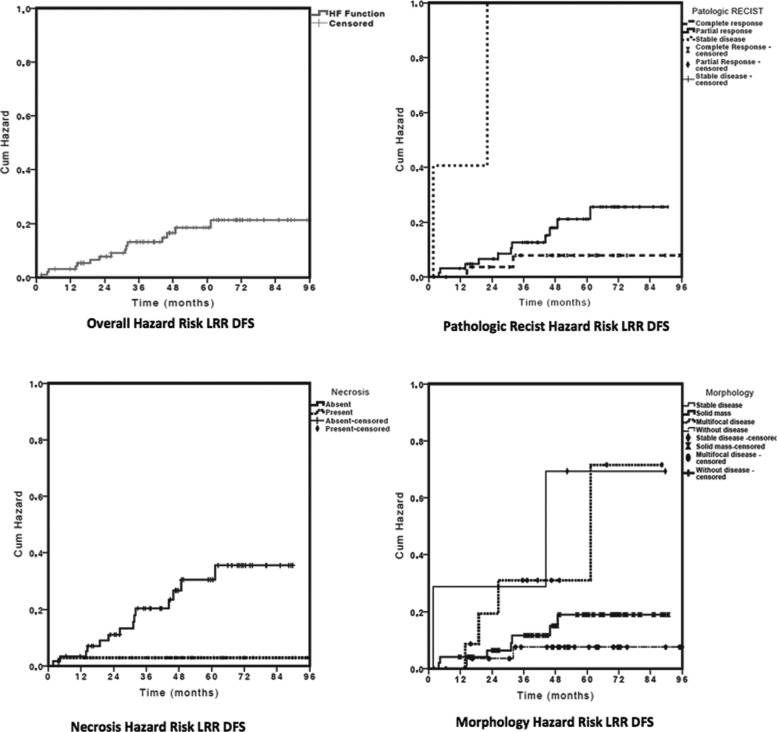
Hazard risk curves of locoregional DFS.

A univariate analysis of the categorical variables relative to locoregional DFS (Table 1) showed that the pathologic RECIST-B response (*p*=0.003), necrosis (*p*=0.008), and morphology were related to locoregional DFS. Neither age (*p*=0.41, risk ratio 1.00, CI 0.98-1.03) nor initial tumor size (*p*=0.27, risk ratio 1.08, CI 0.94-1.23) influenced LRR DFS. The Cox univariate analysis showed that the absence of tumor necrosis at diagnosis increased the LRR 9.33-fold (*p*=0.03), multifocal morphology increased the risk of LRR 6.09-fold (*p*=0.04), and stable disease increased the risk of LRR 9.08-fold (*p*=0.03). However, the RECIST-B pathologic response was the main factor related to locoregional DFS (*p*=0.01) because stable RECIST-B disease increased the risk of LRR 16.93-fold (*p*=0.005). An exploratory Cox multivariate analysis model showed that RECIST-B pathologic response was the only factor related to locoregional DFS. Figure 2 shows the curves related to the hazard risk of locoregional DFS.

## DISCUSSION

NC provides global survival similar to adjuvant chemotherapy, with the added advantage of identifying patients with better prognoses, that is, patients who exhibit pCR in addition to increasing the rates of BTC 18. The primary indication for NC is larger tumors or tumors with higher rates of lymph node involvement. When comparing patients treated with NC and BCS with patients subjected to mastectomy, the former have a lower T-TNM stage at diagnosis; higher rates of pCR; and higher rates of ER-negative, PR-negative, and triple-negative tumors, indicating bias in the analysis of this subgroup. This bias may influence the rates of recurrence and survival 19. In our group of patients, patients who underwent BCS exhibited better survival than the mastectomy group (*p*=0.002), which corroborated previous reports. Bias selection likely occurred based on tumor size, breast-tumor relation, response to NC, and molecular subtype (Table 1) because BCS was performed in patients with smaller tumors, lower clinical TNM stage, and a better response to NC. Because the characteristics differed between groups, we only evaluated patients who underwent BCS. Bleicher et al. evaluated Surveillance, Epidemiology, and End Results (SEER) data from a cohort of 5,685 patients aged >66 years old with tumors >5 cm. Of these patients, 887 (15.6%) underwent BCS, and only 205 (3.6%) received NC. BCS was associated with a lower clinical stage and more NC, but only 101 patients received both NC and BCS, and these patients were not evaluated separately 20. Our study represents one of the largest institutional retrospective cohort studies of LABC treated with NC and BCS 11.

BCS is safe provided that the excision margins are free of disease and this treatment is combined with adjuvant radiotherapy to the breast 21,22. BCS was initially used to treat tumors smaller than 3 cm associated with a 1-cm free margin. These criteria are changing, and smaller margins are currently accepted 23. A meta-analysis of randomized controlled trials showed that BCS is a safe treatment for patients with clinical stage I and II disease and tumors smaller than 5 cm 24. SEER data evaluated for tumors >5 cm indicated that breast cancer-specific survival did not differ between patients who received BCS and patients who underwent a mastectomy, but the women in this study were older, the IBTR and molecular subtype were not evaluated, and few patients received NC 20.

The rate of conservative surgery after NC varies from 37% to 82% 6,7; however, only 1.7% to 28% of patients exhibit LABC 7,8. The LABC candidates who were initially selected were patients without skin or chest wall involvement and who were free of multicentric disease or extensive microcalcifications. They harbored tumors smaller than 5 cm, exhibited favorable tumor localization, had no contraindications for radiotherapy, and had negative margins. Primary inflammatory carcinoma is a contraindication for BCS 25. Patients with N2-3 lymph nodes, residual tumors >2 cm, residual multifocal components, and the presence of lymphovascular embolization should be cautiously assessed because of the higher risk of IBTR 11,26. Therefore, the cutaneous infiltration criteria have become more flexible for localized cutaneous infiltration and the breast/tumor volume ratio, and the initial indications for oncoplastic surgery have been expanded 17. Although the average size of the initial tumors was 5.3 cm (varying from 2 to 8.5 cm) in the present cohort, the margins were disease-free in 100% of cases, with a distance to the tumor of 12.3 mm. In addition, oncoplastic techniques were used in 26.5% of patients, which supports the use of BCS in selected cases of LABC.

The preoperative planning was based on clinical-radiologic data and operative freezing. Two patients were excluded from the cohort because of positive surgical margins, which resulted in conversion to mastectomy.

Diagnostic imaging tests are essential to pretreatment therapeutic planning 3 and were performed before and after the administration of NC in 100% and 87.7% of patients, respectively. Although not shown numerically, a tendency toward the resection of the entire tumor bed before NC was observed in this study. Not all patients who exhibited a complete clinical response 21 reached pCR, and the anatomic-pathologic assessment is not always uniform. In this regard, the “Residual Cancer Burden*”* method renders the resection of the full area necessary prior to NC 27, but it is used in prospective studies. Pathologic sampling interferes with the pathologic results. In the present study, the average number of blocks per surgical specimen was 20, but a consensus for pathologic evaluation was obtained in 2015 28.

Upon the assessment of patients subjected to BCS and radiotherapy, we should consider studies of patients who did not receive NC that demonstrate the long-term safety of BCS. For example, Veronesi 9 assessed tumors smaller than 2 cm and identified a recurrence rate of 8% at 20 years, whereas Fisher (NSAPB-B06), who assessed tumors smaller than 4 cm, reported recurrence rates at 20 years of 14.3% for patients who underwent lumpectomy and breast radiation and 39.2% for patients who did not receive radiation 21. In patients subjected to NC and BCS, this rate was reported to be 14% at 5.8 years 29, 19% at 4.6 years 15, and 21.5% at 20 years 30; however, the assessed tumors differed diagnostically and in their initial staging 19. Therefore, the possibility of new surgical margins remains open for discussion, but case-control studies assessing locally advanced tumors are lacking. NSABP B-27, which assessed patients with T1c-3N0 or T1-3N1M0 disease, was designed to evaluate the addition of taxanes to anthracyclines and reported an average tumor size of 4.4 cm and a 6% IBTR rate at 102 months; however, only 30% of cases exhibited lymph node involvement. In the present cohort, the average tumor size was 5.3 cm, and 87.2% of tumors were larger than 3 cm; 88.9% of patients were diagnosed with stage III disease, 74.5% of patients harbored stage T3-4 disease, and 82.6% of patients had stage N1-3 disease. The IBTR rate was 11.2% at 64.1 months. Although this rate is high, it is lower than the rate reported in a study by Fisher of patients subjected exclusively to lumpectomy without radiotherapy 21. These findings demonstrate the effectiveness of BCS in patients with LABC subjected to NC and adjuvant radiotherapy.

In the assessment of IBTR, we must discriminate true recurrence at the surgical site, ipsilateral second primary tumors, and ipsilateral thoracic wall tumors 31. Although ipsilateral thoracic wall events involving the sternal bone were defined as a distant event in 2014 31, previous studies with long follow-up period did not specify this form of recurrence 32. In the present study, we observed 2 patients with simultaneous IBTR and sternal infiltration, but 1 patient underwent local full-thickness chest wall resection. We opted to consider this case as local recurrence to better compare our results to those of other studies with long follow-up periods. No pattern is associated with the type of local recurrence, but many recurrences are defined as multiple recurrence. Alternatively, recurring tumors may indicate resistance to treatment and subsequent multiple recurrences. In the present study, the LRR rate was 15.3% and consisted of all patients with local recurrence and the 4 patients with locoregional lymph node involvement. This finding corroborates the analysis of the DFS results.

The chi-squared test may be used to calculate recurrence, but we also assessed DFS because recurrence depends on time. Several factors are associated with IBTR and LRR. Better results were observed in patients who showed an early response to treatment 33 and were positive for hormonal receptors 12; poorer outcomes were reported for patients with lymphovascular invasion^11^, residual tumors larger than 2 cm 11, multifocal disease after chemotherapy 11,34, no expression of hormonal receptors, stage III and N2-3 axillary nodal status 15, age ≤40 years old, excision margins ≤2 mm, and S-phase fraction >4% 30.

Few studies have examined a sufficient number of cases to assess the rates of recurrence in patients subjected to NC and BTC 11,33,34. In the present study, which evaluated a large sample over a long follow-up period, the morphologic response, although valid, was not significantly associated with recurrence, whereas the RECIST-B response was shown to have prognostic value in a multivariate analysis. The response to NC can be classified into several categories 35. Chen et al. suggested a morphologic classification of the response, showing that the response correlates with the occurrence of IBTR 11. In the present study, morphologic assessment showed an association, albeit a non-significant one, between the presence of multifocal disease/stable disease and higher rates of IBTR and LRR. The RECIST-B pathologic response (*p*=0.02) was the only variable retained in the multivariate model related to LRR: the risk was 2.85 times higher among patients with a partial response (*p*=0.17) and 16.93 times higher among patients with stable disease (*p*=0005).

In the present cohort, the absence of necrosis in the pretreatment biopsy sample was the only histological factor that was associated with IBTR and LRR. This finding is corroborated by other studies, which detected an association between complete response and the presence of necrosis 36. The absence of necrosis was associated with a 7.00-fold higher rate of IBTR, but this increase was not significant (*p*=0.07). It was also associated with a 9.33-fold higher rate of LRR (*p*=0.03) in univariate analysis. The only significant factor related do LRR in multivariate analysis was the RECIST-B response.

In the assessment of IBTR, other variables, such as the type of tumor fragmentation and the presence of surgical margins, should also be considered. Thus, the rates of IBTR were reported to be 12.7% and 20.3% in the presence and absence of tumor-free margins, respectively 30. In the present study, all patients exhibited tumor-free margins. In a previous study, the presence of multifocal disease increased the risk of IBTR 3.3-fold 12, which is corroborated by this study: multifocal disease increased the rate of IBTR 5.97-fold (*p*=0.04). In addition, the molecular subtypes are related to the rate of recurrence in an adjuvant 37 and neoadjuvant setting 38. Specifically, the rates of recurrence were 0.8% for luminal tumors (ER/PR-positive and HER2-negative), 1.5% for luminal B tumors (ER/PR-positive and HER2-positive), 8.4% for HER2 tumors (ER/PR-negative and HER2 positive), and 7.1% for triple-negative tumors.

A possible limitation of the present study is the retrospective and nonrandomized design in which cases were selected in a continuous manner, that is, based on the feasibility of BCS. Thus, multiple elements influenced the selection of patients, including age, comorbidities, breast-volume ratio, and response to NC. Another limitation of this study is the absence of NC association with trastuzumab, which may affect the pCR, OS, and DFS 39. Because HER2 tumors represent 23.5% 29 of patients, we observed only 3 incidences of local recurrence/LRR in this group and a 0.487-fold reduction in the recurrence rate 39. The addition of trastuzumab would slightly decrease the overall recurrence rate, but this drug was not used by the Brazilian Public Health System at the time of NC treatment.

The present study corroborates the fact that in cases selected by clinical and radiologic findings with a satisfactory response to NC, BCS is feasible and safe for the treatment of locally advanced tumors, provided that the tumor is completely resected, surgical margins are clear, and patients are subjected to complementary multimodal treatment. This finding was corroborated by the occurrence of acceptable rates of local recurrence and LRR.

## AUTHOR CONTRIBUTIONS

Carrara GF participated in the data review, data analysis, and writing of the manuscript. Scapulatempo-Neto C and Abrahão-Machado LF performed the pathological review and reviewed the final version of the manuscript. Brentani MMand FolgueiraMA participated in the study design, data analysis, and review of the final version of the manuscript. Nunes JS participated in the case selection, chemotherapy, submission to the Ethics Committee, and review of the final version of the manuscript. Vieira RA participated in the study design, submission to the Ethics Committee, case selection, surgery, data analysis, and writing of the manuscript.

## Figures and Tables

**Table 1:** Main characteristics of the patients submitted to NC.

Variable	Category	BCS	Mastectomy	All group	*p*
Tumor size	cm	5.23 ± DP 1.64	7.15 ± DP 2.73	6.73 ± DP 2.64	<0.0001
Age at diagnosis	years	48.48 ± DP 11.38	49.76 ± DP 10.55	49.5 ± DP 10.7	0.30
EC TNM	II	13 (13.3%)	10 (2.9%)	23 (5.1%)	<0.0001
	III	85 (86.7%)	341 (97.1%)	426 (94.9%)	
EC-T TNM	T2	25 (25.5%)	18 (5.1%)	43 (9.6%)	
	T3	53 (54.1%)	178 (50.7%)	231 (51.4%)	<0.001
	T4	20 (20.4%)	155 (44.2%)	175 (3.9%)	
EC-N TNM	N0	17 (17.3%)	26 (7.4%)	43 (9.6%)	0.02
	N1	56 (57.1%)	207 (59.0%)	263 (58.6%)	
	N2	22 (22.4%)	105 (29.9%)	127 (28.3%)	
	N3	3 (3.1%)	13 (3.7%)	16 (3.6%)	
Molecular Subtype*	Luminal/ Her-	45 (45.9%)	158 (45.3%)	203 (45.4%)	0.02
	Luminal B/Her +	12 (12.2%)	93 (26.6%)	105 (23.5%)	
	Her 2+	11 (11.2%)	41 (11.7%)	52 (11.6%)	
	Triple negative	30 (30.6%)	57 (16.3%)	87 (19.5%)	
NC response	Non pCR	71 (72.4%)	301 (85.8%)	372 (82.9%)	<0.0001
	pCR	27 (27.6%)	50 (14.2%)	77 (17.1%)	

* cases with missing data

**Table 2:** Univariate analysis of factors related to local and locoregional recurrence -free survival.

Category	Variable	n (%)	Local 60 months DFS	*p*	Locoregional 60 months DFS	*p*
**Pre-operative**						
EC TNM	II	13 (13.3)	77.1	0.54	67.5	0.37
	III	85 (86.7)	89.5		82.8	
ECT - TNM	T2	25 (25.5)	78.6	0.53	66.8	0.23
	T3	53 (54.1)	89.2		85.6	
	T4	20 (21.4)	95.0		95.0	
ECN - TNM	N0	17 (17.3)	100.0	0.71	100.0	0.40
	N1	56 (57.1)	85.2		81.0	
	N2-3	25 (25.5)	87.2		77.8	
Tumor marking	Absent	75 (76.5)	86.0	0.68	79.9	0.37
	Present	23 (23.5)	95.0		95.0	
Histologic type	CDI	90 (89.8)	89.5	0.13	84.1	0.31
	CLI+ other	8 (8.2)	70.0		70.0	
Nottinghan grade*	G1+2	56 (56.4)	85.1	0.29	76.4	0.07
	G3	40 (41.7)	91.2		91.2	
Necrosis*	Absent	60 (61.2)	81.9	0.039	73.7	0.008
	Present	36 (37.5)	97.1		97.1	
Peritumoral Infiltration*	Slight	56 (57.2)	89.9	0.80	85.2	0.68
	Moderate/intense	10 (40.8)	85.0		79.4	
Lymphatic embolization*	Absent	85 (88.5)	86.6	0.29	82.1	0.75
	Present	11 (11.5)	100.0		90.9	
ER	Negative	45 (45.9)	86.9	0.50	82.7	0.90
	Positive	53 (54.1)	88.9		83.4	
PR	Negative	52 (53.1)	88.6	0.80	86.7	0.49
	Positive	46 (46.9)	86.9		78.8	
Her2	Positive	23 (23.5)	86.1	0.79	80.0	0.41
	Negative	75 (76.5)	88.8		89.0	
Molecular Subtype	Luminal / Her -	45 (45.9)	85.5	0.63	79.0	0.40
	Luminal B Her+	12 (12.2)	91.7		91.7	
	Her2	11 (11.2)	78.8		79.0	
	Triple negative	30 (30.6)	93.2		89.8	
**Postoperative**						
Oncoplastic surgery	Absent	72 (73.5)	85.0	0.50	79.7	0.52
	Present	26 (26.5)	96.0		92.0	
RECIST-B	Complete response	31 (31.6)	92.6	0.16	92.7	0.003
	Partial response	64 (64.3)	86.9		80.6	
	Stable disease	3 (3.1)	66.7		33.3	
Morphology	Solid mass	50 (51.0)	89.9	0.04	82.7	0.03
MDA 11	Multifocal disease	13 (13.3)	77.3		69.5	
	Without disease	31 (31.6)	92.0		92.1	
	Stable disease	4 (4.1)	75.0		50.0	
pCR/ NSABP	Absent	71 (72.4)	87.3	0.58	80.6	0.28
	Present	27 (27.6)	90.4		90.4	

DFS = disease free survival; pCR = pathologic complete response;

* 2 cases missing data

**Table 3:** Cox analysis of factors related to local and locoregional recurrence-free survival.

Variable	Category	OR	CI	*p* factor	*p* general
**Local recurrence (IBTR)**					
Necrosis	Present	1.00	Ref		0.07
	Absent	7.00	0.85-57.19	-	
Morphology	Without disease	1.00	Ref.		0.08
MDA 11	Solid mass	1.28	0.23-6.98	0.78	
	Stable disease	4.60	0.42-50.79	0.21	
	Multifocal disease	5.97	1.09-32.70	0.04	
**Locoregional recurrence (LRR)**					
RECIST-B	Complete response	1.00	Ref.		0.01
	Partial response	2.85	0.63-12.85	0.17	
	Stable disease	16.93	2.37-120.84	0.005	
Necrosis	Present	1.00	Ref.		0.03
	Absent	9.33	1.23-71.03		-
Morphology	Without disease	1.00	Ref.		0.06
MDA 11	Solid mass	2.25	0.47-10.86	0.31	
	Multifocal disease	6.09	1.11-33.40	0.04	
	Stable disease	9.08	1.28-64.51	0.03	
Nottinghan grade	G1+2	1.00	Ref.		0.08
	G3	0.33	0.09-1.16		-

OR = odds ratio; CI = confidence interval; Ref. = Reference
